# A Four-Gene-Based Prognostic Model Predicts Overall Survival in Patients With Cutaneous Melanoma

**DOI:** 10.3389/fonc.2021.639874

**Published:** 2021-03-24

**Authors:** Xiaoxia Tong, Xiaofei Qu, Mengyun Wang

**Affiliations:** ^1^ Cancer Institute, Fudan University Shanghai Cancer Center, Shanghai, China; ^2^ Department of Oncology, Shanghai Medical College, Fudan University, Shanghai, China

**Keywords:** prognosis, cutaneous melanoma, risk score, gene signature, survival

## Abstract

**Background:**

Cutaneous melanoma (CM) is one of the most aggressive cancers with highly metastatic ability. To make things worse, there are limited effective therapies to treat advanced CM. Our study aimed to investigate new biomarkers for CM prognosis and establish a novel risk score system in CM.

**Methods:**

Gene expression data of CM from Gene Expression Omnibus (GEO) datasets were downloaded and analyzed to identify differentially expressed genes (DEGs). The overlapped DEGs were then verified for prognosis analysis by univariate and multivariate COX regression in The Cancer Genome Atlas (TCGA) datasets. Based on the gene signature of multiple survival associated DEGs, a risk score model was established, and its prognostic and predictive role was estimated through Kaplan-Meier (K-M) analysis and log-rank test. Furthermore, the correlations between prognosis related genes expression and immune infiltrates were analyzed *via* Tumor Immune Estimation Resource (TIMER) site.

**Results:**

A total of 103 DEGs were obtained based on GEO cohorts, and four genes were verified in TCGA datasets. Subsequently, four genes (*ADAMDEC1, GNLY, HSPA13*, and *TRIM29*) model was developed by univariate and multivariate Cox regression analyses. The K-M plots showed that the high-risk group was associated with shortened survival than that in the low-risk group (*P* < 0.0001). Multivariate analysis suggested that the model was an independent prognostic factor (high-risk vs. low-risk, HR= 2.06, *P* < 0.001). Meanwhile, the high-risk group was prone to have larger breslow depth (*P*< 0.001) and ulceration (*P*< 0.001).

**Conclusions:**

The four-gene risk score model functions well in predicting the prognosis and treatment response in CM and will be useful for guiding therapeutic strategies for CM patients. Additional clinical trials are needed to verify our findings.

## Introduction

Cutaneous melanoma (CM) accounts for over 74% of skin cancer related death each year (1), which makes it one of the most malignant cancers, with tremendously poor prognosis (2, 3). The incidence of CM has continued to increase annually. Although tremendous efforts toward early detection and therapeutics were made, advanced stage melanoma patients still exhibit disappointing prognosis with 5-year overall survival rate ranging from 45% for stage III to 18% for stage IV (4, 5).

Cutaneous melanoma is a highly heterogeneous tumor, in terms of clinical and complicated molecular (5). Several clinical features, such as age, gender, stage, ulceration and breslow thickness have been shown to be the important clinicopathological characteristics for predicting the outcome of CM patient (6). However, due to the high potentiality for CM metastasis, the prognosis remains poor. Molecular biomarkers are important in guiding treatment selection and predicting outcome in tumor patients (7–9). For example, the 21-gene recurrence score assay is prognostic for women with node-negative, estrogen-receptor-positive breast cancer treated with tamoxifen (10). Although hundreds of studies have explored the prognostic value of molecular markers, there is still no recommended molecular marker to predict CM prognosis.

In the current study, we were devoted to exploring new biomarkers and establishing a risk score model to predict prognosis, aiming to provide appropriate therapeutic methods for CM patients.

## Materials and Methods

### Gene Expression Omnibus (GEO) Datasets Collection and Enrichment Analysis

Gene expression raw microarray cell intensity (CEL) profiles of CM were evaluated in three independent datasets from the GEO database (accession number: GSE7553, GSE46517, and GSE15605), which included 57 tumor tissue samples and three normal skin samples; 85 tumor tissue samples and eight normal skin samples; 60 tumor tissue samples and six normal skin samples, respectively. The microarray data GSE65904 containing 214 patients was downloaded to verify our risk model. Four patients were deleted due to lack of follow-up information. When more than one probe matched the same gene ID, the mean expression value of the gene was used for our study.

### The Cancer Genome Atlas (TCGA) Dataset

The TCGA CM dataset, containing 459 tumor samples which included raw counts of RNAseq expression data and clinicopathological characteristics were obtained from cBioPortal website. The TCGA dataset was randomly divided into two parts: the training cohort and the validation cohort.

### Identification of Common Differential Expression Genes (DEG)

The GSE7553, GSE46517, and GSE15605 expression profiles were normalized and the DEG were calculated using the LIMMA package. In this study, Gene sets with False Discovery Rate (FDR) < 0.05 and with the threshold of |logFC|>1 were defined as DEGs. All the data processing and normalization were performed using the R software.

### Identification and Selection of Prognosis-Related Genes

Univariate and multivariate Cox regression analyses model were commonly employed in survival analysis. Genes were considered significant when the *P* value were <0.05 in the univariate and multivariate Cox regression analysis based on training and validation cohorts. These genes were used to construct the risk model. The fitness of the models was compared based on Akaike information criterion (AIC) and the lowest value of AIC provided the sensitivity and specificity. Subsequently, four genes (*ADAMDEC1, GNLY, HSPA13*, and *TRIM29*) were selected.

### Construction and Assessment of Risk Score System

Based on the prognosis associated genes, a risk score model was constructed for the CM patients. Each gene was added one at a time in the risk score system and the risk score for each patient was calculated as the sum of each gene’s score as follows:

$$\text{Risk score}=\beta \text{gene}1{}^{*}\text{Exp gene}1+\beta \text{gene}2{}^{*}\text{Exp gene}2+\;\cdot \cdot \cdot \;+\beta \text{gene}(n){}^{*}\text{Exp gene}(\text{n})$$

In this formula, βgene(n) represents the coefficient of each gene from univariate Cox regression analysis, and Exp gene(n) displays the expression of each gene.

Then all TCGA patients were separated into high and low-risk subgroups according to the optimal cut-off value of risk score. The optimal cut-off value of risk score was determined by the time-dependent receiver operating characteristic (ROC) curve using “survivalROC” package. To compare the survival time difference between the low- and high-risk group, K-M curve was produced by the “Survminer” package using the log-rank test. The predictive accuracy of this risk score model was determined by time-dependent ROC curve analysis. The area under the curve (AUC) was calculated to measure the predictive ability of the gene signature for clinical outcomes.

### Immune Infiltration Analysis

The abundance of tumor infiltrating immune cells in CM was predicted using the Tumor Immune Estimation Resource (TIMER) algorithm. The correlation between prognostic gene expression and the abundance of different immune cells, including CD8+ T cells, CD4+ T cells, macrophages, B cells, neutrophils, and dendritic cells was measured using the Spearman’s test. All hypothetical tests were two-sided and *P* values < 0.05 were considered statistically significant.

## Results

### Screening of DEG

**Figure 1:**
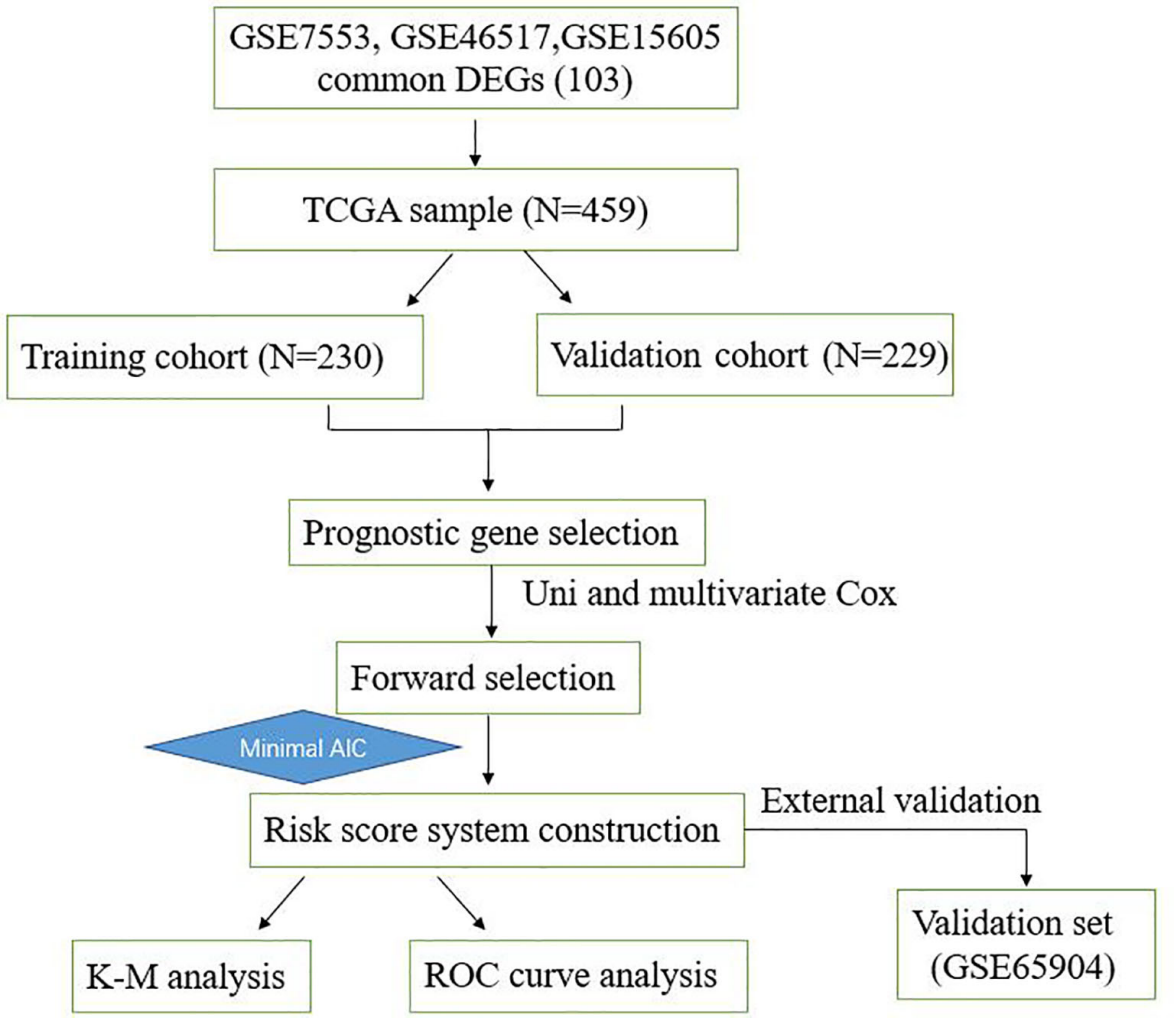
Overall workflow describing the process used to develop and validate the prognostic model to predict prognostic outcomes.

**Figure 2:**
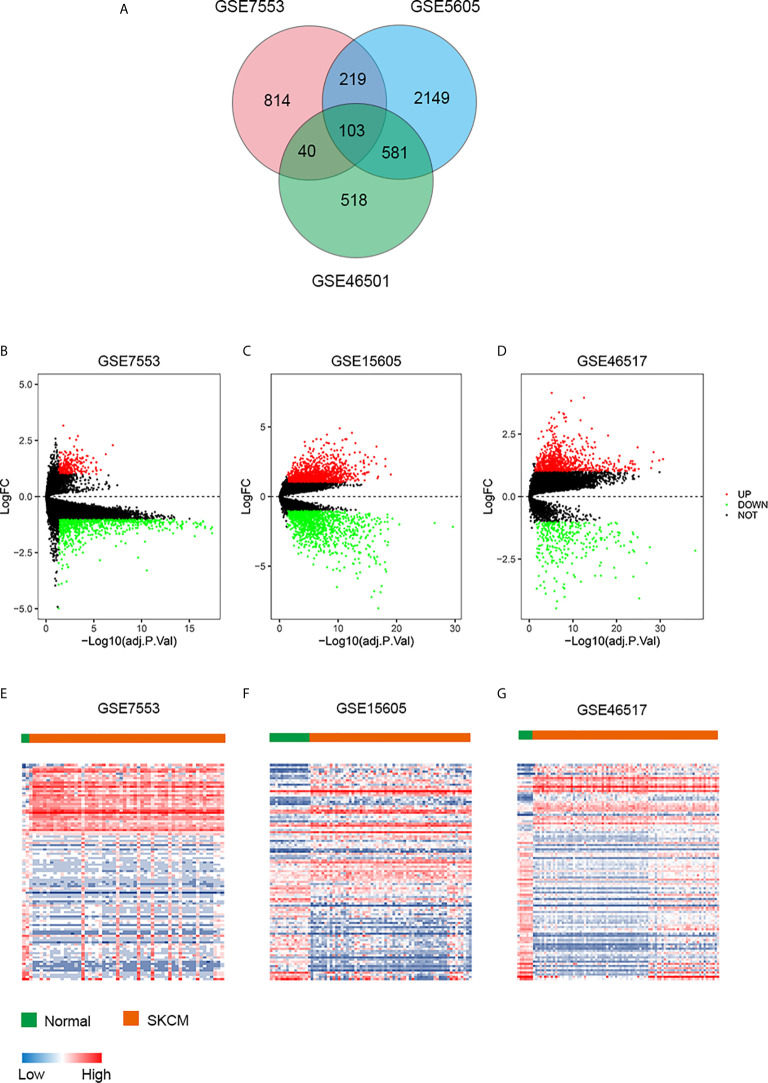
DEGs in three data sets. **(A)** Venn diagram of DEGs. **(B–D)** The volcano plots visualize the DEGs in GSE7553, GSE15605, and GSE46517, respectively. The red nodes represent upregulated genes. The green nodes represent downregulated genes. **(E–G)** Heatmap of the top 103 DEGs according to the value of |logFC| > 1 and FDR < 0.05. The color in heat maps from blue to red shows the progression from low expression to high expression. logFC, log fold change.

To describe our study more clearly, a flow chart of the analysis procedure was developed (**Figure 1**). After the analyses of GSE7553, GSE46517, and GSE15605 data sets, DEGs were identified and selected. The overlap among three data sets included 103 DEGs was shown in the Venn diagram (**Figure 2A**). The volcano plots and heatmap of each data set are shown in **Figures 2B–G**.

### Construction of Risk Score System

We conducted univariate and multivariate Cox regression to investigate the correlation of the DEGs with the overall survival of TCGA CM patients in training, validation and total cohort. Basic characteristics of the patients are shown in **Table 1**. The result revealed that *GNLY, DFNA28, ADAMDEC1, ALOXE3, EFNA3, EPN3, EVPL, FERMT1, HSPA13, JAG2, RAPGEFL1, SULT2B1, TGM3*, and *TRIM29* were significant prognostic factors. Furthermore, in order to select the best performance efficacy predictive model with the lowest AIC value, we performed the stepwise multivariate Cox regression analysis to identify independent predictors for overall survival of total TCGA CM patients. Finally, four prognosis−associated genes *(GNLY, ADAMDEC1, HSPA13*, and *TRIM29*) were selected for constructing the risk score system (**Table 2**). The formula was as follows:

$$\begin{array}{c} Riskscore=\left(-0.101\right){}^{*}ExpADAMDEC1+\left(-0.091\right){}^{*}ExpGNLY+\\ \left(-0.284\right){}^{*}ExpHSPA13+0.102{}^{*}ExpTRIM29 \end{array}$$

**Table 1:** Basic characteristics of TCGA CM patients.

Characteristics	Groups	Total (N=459)		Training cohort (N=229)		Validation cohort (N=230)	
		No	%	No	%	No	%
Age	≤58	233	50.7	116	50.7	117	50.9
	>58	226	49.3	113	49.3	113	49.1
Sex	Female	175	38.1	82	35.8	93	40.4
	Male	284	61.9	147	64.2	137	59.6
Metastasis	No	410	89.3	209	91.3	201	87.4
	Yes	23	5	9	3.9	14	6.1
	missing	26	5.7	11	4.8	15	6.5
Ulceration	No	145	31.6	70	30.6	75	32.6
	Yes	165	35.9	92	40.2	73	31.7
	Missing	149	32.5	67	29.2	82	35.7
Pathologic Stage	0	6	1.3	3	1.3	3	1.3
	I	77	16.8	40	17.5	37	16.1
	II	139	30.3	73	31.8	66	28.7
	III	169	36.8	81	35.4	88	38.3
	IV	22	4.8	9	4	13	5.6
	Missing	46	10	23	10	23	10
Tumor Site	Trunk	166	36.2	86	37.6	80	34.8
	Extremities	194	42.3	100	43.7	94	40.9
	Head and neck	35	7.6	15	6.5	20	8.7
	Missing	64	13.9	28	12.2	36	15.6
Breslow thickness (mm)	≤2	136	29.6	73	31.9	63	27.4
	2–5	113	24.6	52	22.7	61	26.5
	>5	105	22.9	59	25.8	46	20
	Missing	105	22.9	45	19.6	60	26.1
Chemotherapy	No	323	70.4	153	66.8	170	73.9
	Yes	88	19.2	49	21.4	39	17
	Missing	48	10.4	27	11.8	21	9.1
Radiotherapy	No	341	74.3	170	74.2	171	74.3
	Yes	73	15.9	34	14.9	39	17
	Missing	45	9.8	25	10.9	20	8.7

**Table 2:** Univariate and multivariate analysis of prognosis genes for TCGA CM.

Training cohort	Univariate analysis			Multivariate analysis		
Genes	coef	HR (95%CI)	*P*	coef	HR (95%CI)	*P*
ADAMDEC1	−0.098	0.906 (0.848–0.969)	0.004	−0.099	0.906 (0.845–0.971)	0.005
D2S69E	−0.084	0.919 (0.847–0.998)	0.043	−0.089	0.915 (0.839–0.998)	0.045
HSPA13	−0.346	0.708 (0.594–0.844)	0.000	−0.251	0.778 (0.648–0.936)	0.007
TRIM29	0.088	1.092 (1.033–1.154)	0.002	0.074	1.077 (1.017–1.141)	0.011
Validation cohort	Univariate analysis			Multivariate analysis		
Genes	coef	HR (95%CI)	*P*	coef	HR (95%CI)	*P*
ADAMDEC1	−0.102	0.903 (0.852–0.958)	0.000	−0.113	0.893 (0.834–0.956)	0.001
D2S69E	−0.099	0.905 (0.843–0.971)	0.006	−0.092	0.090 (0.844–0.986)	0.021
HSPA13	−0.232	0.793 (0.637–0.987)	0.038	−0.227	0.797 (0.641–0.991)	0.042
TRIM29	0.112	1.119 (1.066–1.174)	0.000	0.101	1.106 (1.052–1.164)	0.000
Total	Univariate analysis			Multivariate analysis		
Genes	coef	HR (95%CI)	*P*	coef	HR (95%CI)	*P*
ADAMDEC1	−0.101	0.905 (0.866–0.945)	0.000	−0.108	0.898 (0.857–0.940)	0.000
D2S69E	−0.091	0.913 (0.865–0.963)	0.000	−0.093	0.911 (0.862–0.964)	0.000
HSPA13	−0.284	0.753 (0.657–0.864)	0.000	−0.264	0.768 (0.669–0.882)	0.000
TRIM29	0.102	1.108 (1.068–1.148)	0.000	0.091	1.095 (1.055–1.136)	0.000

**Figure 3:**
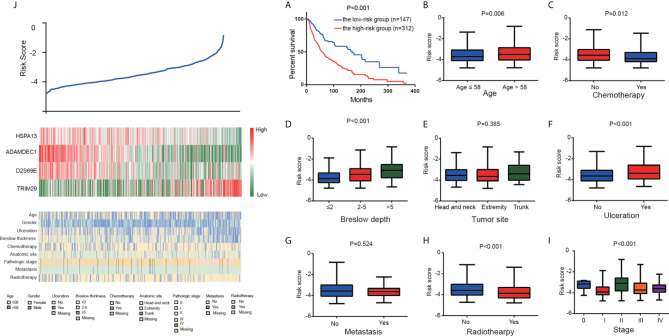
The four-gene signature-derived risk score. **(A)** Kaplan–Meier overall survival analysis among TCGA CM patients stratified by risk score. Association between the risk model and different clinical characteristics. **(B)** Age. **(C)** Chemotherapy. **(D)** Breslow depth. **(E)** Tumor site. **(F)** Ulceration. **(G)** Metastasis. **(H)** Radiotherapy. **(I)** Stage. **(J)** The value of risk score (top), The corresponding expression of four genes (middle), and the associated clinicopathological parameters (bottom).

To evaluate the prognostic significance of the risk score, K-M plot of high and low risk CM patients were conducted. According to the optimal cut-off value of risk score, the patients in the total TCGA cohort were classified into high (312 patients) and low (147 patients) risk groups. Compared to the high-risk group with the median OS time of 27.76 months, the low-risk group with the median OS time of 56.8 months had a higher survival ratio (P<0.001; **Figure 3A**).

Furthermore, we analyzed the correlation between risk score and clinicopathological characteristics, which showed that high risk score was positively associated with elder age, ulceration, and breslow depth. Patients who received chemotherapy and radiotherapy prone to low-risk (**Figures 3B–J**).

### Stratification Analysis

**Figure 4:**
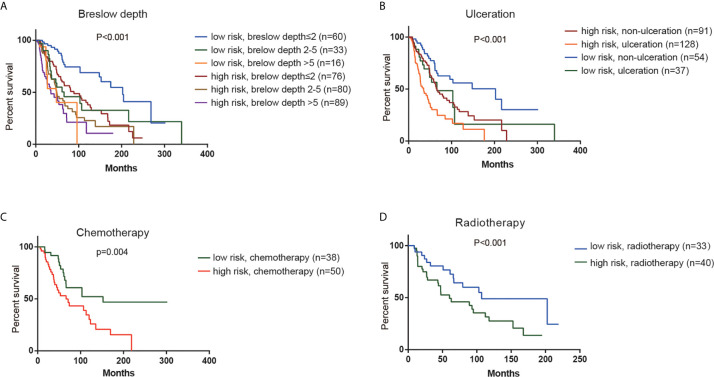
Stratification analysis. Kaplan–Meier overall survival analysis of TCGA CM patients stratified by risk scores combined with **(A)** Breslow depth. **(B)**. Ulceration. **(C)** Chemotherapy. **(D)** Radiotherapy.

According to K-M analysis, CM patients with high risk score and larger breslow depth had the worst outcomes (**Figure 4A**), and CM patients with the ulceration and high- risk score had a shorter survival time than those with the non-ulceration group (**Figure 4B**). Furthermore, high risk score was also associated with poor prognosis in CM patients treated with chemotherapy or radiotherapy (**Figures 4C, D**), indicating that the risk score could predict the therapeutic reaction.

### Survival Predictive Model Based on Clinical Factors Alone or Their Combination With Risk Score

**Figure 5:**
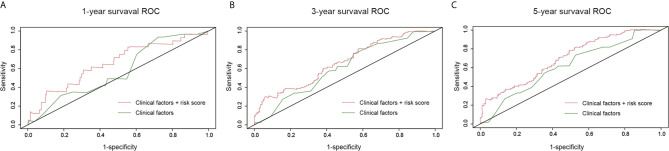
Survival prediction model under the comparison of clinical factors versus the combination of risk score and clinical factors. **(A)** One-year survival receiver operating characteristic curves (ROC); **(B)** 3-year survival ROC; **(C)** 5-year survival ROC.

We constructed a survival prediction model to identify whether risk score in the presence of clinical factors to better discriminate survival of CM patients. Compared with the model with clinical factors alone, the model with addition of the risk score improved the sensitivity and specificity of discriminating 1-year (AUC, 0.57 to 0.66, **Figure 5A**), 3-year (AUC, 0.61 to 0.66, **Figure 5B**), and 5-year survival (AUC, 0.61 to 0.70, **Figure 5C**). When the model had both the risk score and clinical factors, its predictive ability for survival was greater [Concordance index (C-index) =0.66] than that with clinical factors alone (C-index=0.59).

### External Validation of the Model in GSE65904

**Figure 6:**
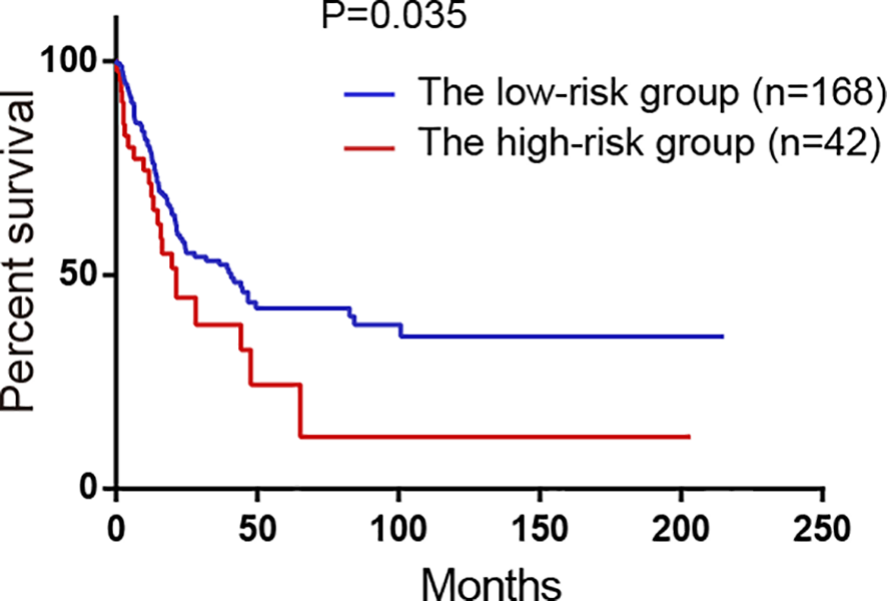
Survival analysis of the high-risk group and the low-risk group divided by the model in GSE65904 validation set. All 214 patients were classified into two groups: the high-risk group and the low-risk group by the optimal cut-off value of risk scores.

GSE65904 dataset was used to validate the prediction performance of the model and each patient’s risk score was calculated according to the formula of the model. All patients were divided into two groups: the high-risk group and the low-risk group by the optimal cut-off value of risk score. The K-M curve revealed significant difference in overall survival between groups in GSE65904. High-risk group had markedly poorer outcome than low-risk group with P < 0.05 in **Figure 6**.

### The Association Between Prognosis Related Gene and Immune Markers

In order to detect the correlation between prognosis related gene and the immune infiltration level, we concentrated particularly on the relationship between prognosis related gene and immune markers of various immune cells in CM using the TIMER database. There was a positive correlation between ADAMDEC1 expression and the dendritic cell (Cor=0.67, *p*=4.72e−59), neutrophils (Cor=0.652, *p*=3.99e−56), CD8+

**Figure 7:**
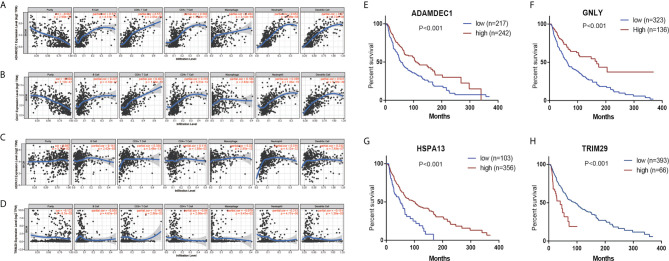
The correlation between prognostic genes expression and immune cell infiltration in CM (TIMER database). The correlation between the abundance of immune cell and the expression of **(A)** ADAMDEC1; **(B)** GNLY; **(C)** HSPA13; **(D)** TRIM29. Prognostic values of **(E)** ADAMDEC1, **(F)** GNLY, **(G)** HSPA13, **(H)** TRIM29 in TCGA CM.

T cells (Cor=0.572, *p*=2.05e−39), macrophages (Cor=0.404, *p*=3.12e−19), CD4+ T cells (Cor=0.385, *p*=3.45e−17), B cells (Cor=0.371, *p*=4.51e−16). Similar results were obtained for GNLY and HSPA13 (**Figures 7A–C**). While, the correlation between TRIM29 and immune infiltration is not obvious (**Figure 7D**). According to K-M analysis, high *ADAMDEC1, HSPA13*, and *GNLY* expression was significantly correlated with better prognosis, while high TRIM29 expression was markedly correlated with poor prognosis (**Figures 7E–H**).

## Discussion

In present study, we selected and constructed a four-gene based risk score model for CM. We analyzed GSE7553, GSE46517, and GSE15605 data sets, 103 DEGs were identified and selected. Subsequently, univariate and multivariate COX regression were employed for the key genes. Fourteen genes (*GNLY, DFNA28, ADAMDEC1, ALOXE3, EFNA3, EPN3, EVPL, FERMT1, HSPA13, JAG2, RAPGEFL1, SULT2B1, TGM3*, and *TRIM29*) were finally identified to be the prognostic genes. Here we adopted stepwise multivariate Cox regression analysis to select the best performance efficacy predictive model with the lowest AIC value. Finally, a four-gene based model including *GNLY, ADAMDEC1*, *HSPA13*, and *TRIM29* was successfully developed.

Furthermore, in order to evaluate the prognostic significance of the new risk model, we performed log-rank test and the ROC curve analysis to investigate association between the model and clinical parameters. As we expected, the high-risk cohort was correlated with poor outcome and was tend to larger breslow depth and ulceration.

For our prognosis related genes, researchers have revealed that some of them may be crucial in cancer development, including CM. For instance, ALOXE3, which encodes arachidonate lipoxygenase3, can serve as a potential predictive biomarker for colon adenocarcinoma patients. Low expression of ALOXE3 had a favorable prognosis of COAD (11). Gómez-Maldonado et al. identified EFNA3, a member of the ephrin type A ligands, is induced by hypoxia-inducible factor in human tumors and this induction is predictive of poor prognosis and increased risk of metastasis in breast cancer patients (12). EPN3 expression is upregulated in wounded epithelial tissues and it can drive breast tumorigenesis by increasing E-cadherin endocytosis, EPN3 is overexpressed in 40% of breast cancers and its overexpression is an independent predictor of distant metastasis (13, 14).. Envoplakin (EVPL) is a protein component of desmosomes and the DNA variant in intron of EVPL (rs2071194) has been found associated with papillary and follicular thyroid cancer risk (15). FERMT1, as an oncogene, promotes the degradation of IκBα, thereby activating NF-κB signaling and promoting gastric cancer (16). JAG2 is one of Notch ligands, which recently appear to exert various carcinogenesis. JAG2 expression significantly correlates with angiogenic processes and vascular development in breast cancer, and is induced at the transcriptional level in hypoxic tumor cells. The oncogene c-myc can also modulate JAG2 expression under hypoxic conditions (17). In 2013, Takahashi et al. reported that RAPGEFL1 was highly methylated in some ESCC cell lines and RAPGEFL1 could regulate by most miRNAs. Therefore, RAPGEFL1 may be the potential pathogenic genes for ESCC (18). TGM3 could affect epithelial-mesenchymal transition, play an essential role in tumorigenesis and progression. It might serve as a useful biomarker and potential therapeutic target for hepatocellular carcinoma treatment (19).

Several genes in our risk model had been investigated in immune response. TRIM29, a member of the tripartite interaction motif (TRIM) family of proteins, functions as a negative regulator of innate immune response. Studies have shown that knockdown of TRIM29 in airway epithelial cells enhances type I interferon production (20). TRIM29 is also recognized as an oncogene, and elevated gene expression in multiple tumors such as colorectal cancer and bladder cancer and so on (21). But the function of TRIM29 in cutaneous melanoma remained still unknown. Elizabeth et al. discovered that ADAMDEC1, an orphan ADAM-like metalloprotease, is expressed in the immune system, by dendritic cells and macrophages. *In vitro*, the expression of ADAMDEC1 was significantly elevated in M1 but not M2 macrophages. More research is needed to determine the associations between ADAMDEC1 and immune response and associations with survival for cancers (22). Granulysin (GNLY) is a cytolytic apoptotic molecule highly expressed in activated immune cells, particularly human cytotoxic T lymphocytes (CTLs) and natural killer (NK) cells (23). GNLY functions as a lytic molecule to carry out lysis or apoptosis product in target cells, including tumor cells or cells infected by pathogens. GNLY can also activate antigen-presenting cells through TLR4 (24). Multiple publications have confirmed the anti-tumor activity of GNLY (25–29). Ya-Wen reported that the serum level of GNLY was negatively correlated with the proliferation of transplanted tumor cells in HIS mice (30). All gene in this risk model are firstly studied in cutaneous melanoma.

To sum up, our research results indicate that the four-gene prognostic model is a reliable tool for predicting the overall survival of CM, it may be useful for guiding therapeutic strategies to improve the clinical outcome of melanoma patients. The low- risk group should avoid some unnecessary treatment to reduced drug toxicities, and high-risk group can receive other intensive treatment. For clinical application, more clinical studies are needed to further verify the prognostic and predictive significance of the risk score model.

## Conclusions

In conclusion, the new risk score system functions well in predicting the prognosis and treatment response in CM patients, with the potential to optimize treatment options. More studies are needed to explore the biological function of these four genes in CM progression and to further verify the prognostic value of the model for clinical practice.

## Data Availability Statement

The original contributions presented in the study are included in the article/supplementary material. Further inquiries can be directed to the corresponding author.

## Author Contributions

This work was carried out in collaboration with all authors. MW designed the theme of the article. XT wrote and XQ reviewed the article. All authors contributed to the article and approved the submitted version.

## Conflict of Interest

The authors declare that the research was conducted in the absence of any commercial or financial relationships that could be construed as a potential conflict of interest.
